# Prevalence and risk factors of *Strongyloides stercoralis* in Takeo Province, Cambodia

**DOI:** 10.1186/1756-3305-7-221

**Published:** 2014-05-12

**Authors:** Virak Khieu, Fabian Schär, Hanspeter Marti, Philipp J Bless, Meng Chuor Char, Sinuon Muth, Peter Odermatt

**Affiliations:** 1 National Centre for Parasitology, Entomology and Malaria Control, Ministry of Health, Phnom Penh, Cambodia; 2 Department of Epidemiology and Public Health, Swiss Tropical and Public Health Institute, Basel, Switzerland; 3 University of Basel, Basel, Switzerland; 4 Medical and Diagnostics Department, Swiss Tropical and Public Health Institute, Basel, Switzerland

**Keywords:** *Strongyloides stercoralis*, Prevalence, Risk factors, Clinical manifestation, Cambodia

## Abstract

**Background:**

The threadworm *Strongyloides stercoralis*, the most neglected helminth, affects an estimated 30-100 million people worldwide. Information on *S. stercoralis* infection is scarce in tropical and sub-tropical resource poor countries, including Cambodia. We determined *S. stercoralis* infection prevalence and risk factors for infection in the general population in Southern Cambodia.

**Methods:**

A cross-sectional study was carried out between January and April 2011 among 2,861 participants living in 60 villages of Takeo province, using Koga-agar plate culture, the Baermann technique and the Kato-Katz technique on a single stool sample.

**Results:**

Eight intestinal helminth species were diagnosed. Hookworm (31.4%) and *S. stercoralis* (21.0%) occurred most frequently. Prevalence of *S. stercoralis* infection increased with age. In all age groups a higher prevalence was found among males than among females (OR: 1.7; 95% CI: 1.4 – 2.0; *P* < 0.001). Participants who had a latrine at home were significantly less frequently infected with *S. stercoralis* than those who did not (OR: 0.7; 95% CI: 0.4 – 0.8; *P* = 0.003). Muscle pain (OR: 1.3; 95% CI: 1.0 – 1.6; *P* = 0.028) and urticaria (OR: 1.4; 95% CI: 1.1 – 1.8; *P* = 0.001) were significantly associated with *S. stercoralis* infection.

**Conclusions:**

*S. stercoralis* is highly prevalent among the general Cambodian population and should no longer be neglected. Access to adequate diagnosis and treatment is urgently needed.

## Background

*Strongyloides stercoralis*, a soil-transmitted nematode, is arguably the most neglected tropical disease [1], yet an estimated 30 – 100 million people are infected worldwide [2]. Despite its frequency, epidemiological data on prevalence and geographical variations are largely lacking [3]. The prevalence of *S. stercoralis* is often underestimated, as most diagnostic methods used have a low sensitivity for *S. stercoralis*[4-6]. Furthermore, in resource poor countries, environmental conditions and poor hygiene behaviour favour transmission. For Cambodia, only a few reports are available, giving prevalences ranging from 2.6% to 24.4% [6-10].

To date, there is no universally agreed upon gold standard for diagnosing *S. stercoralis*. Molecular and serological methods have been reported as promising diagnostic tools, yet these techniques require further validation or further development [11]. Therefore, definitive diagnosis relies on the detection of larvae in stool specimens [4]. The Baermann technique [12] and Koga-agar plate (KAP) culture [13] are the coprological diagnostic methods most often employed. The sensitivity and specificity of either technique alone is not satisfactory [6,14-16]. Thus, to increase the likelihood of detecting *S. stercoralis*, combined use of these diagnostic tests has been suggested [6].

*S. stercoralis* infection is acquired by infective filariform larvae (L_3_), originating from contaminated soil [17], directly penetrating the skin. The clinical presentation of strongyloidiasis is extremely variable, ranging from asymptomatic patients to patients with gastrointestinal symptoms (e.g., abdominal pain, diarrhoea) and urticaria [18], to disseminated infections with mortality rates as high as 87% [19].

The present cross-sectional study aimed to determine the prevalence, risk factors and associated clinical manifestations of *S. stercoralis* infection in a random population sample living throughout 60 villages of Takeo province, southern Cambodia.

## Methods

### Ethical considerations

The study was approved by the National Ethics Committee for Health Research, Ministry of Health of Cambodia (NECHR # 185, dated 20 December 2010) and by the Ethics Committee of the Cantons of Basel-Stadt and Baselland, Switzerland (EKBB #14/11, 13 January 2011). All participants were informed in Khmer language about the purpose and procedures of the study. Written informed consent was obtained from all participants prior to enrolment. For participants aged 1 to 18 years, consent was obtained from the parents, legal guardian or appropriate literate substitutes.

All participants infected with *S. stercoralis* were treated with a single oral dose of ivermectin (200 μg/kg body weight) [20]. All other parasitic infections were treated according to the guidelines of the National Helminth Control Program of Cambodia [21].

### Study design, area and population

**Figure 1:**
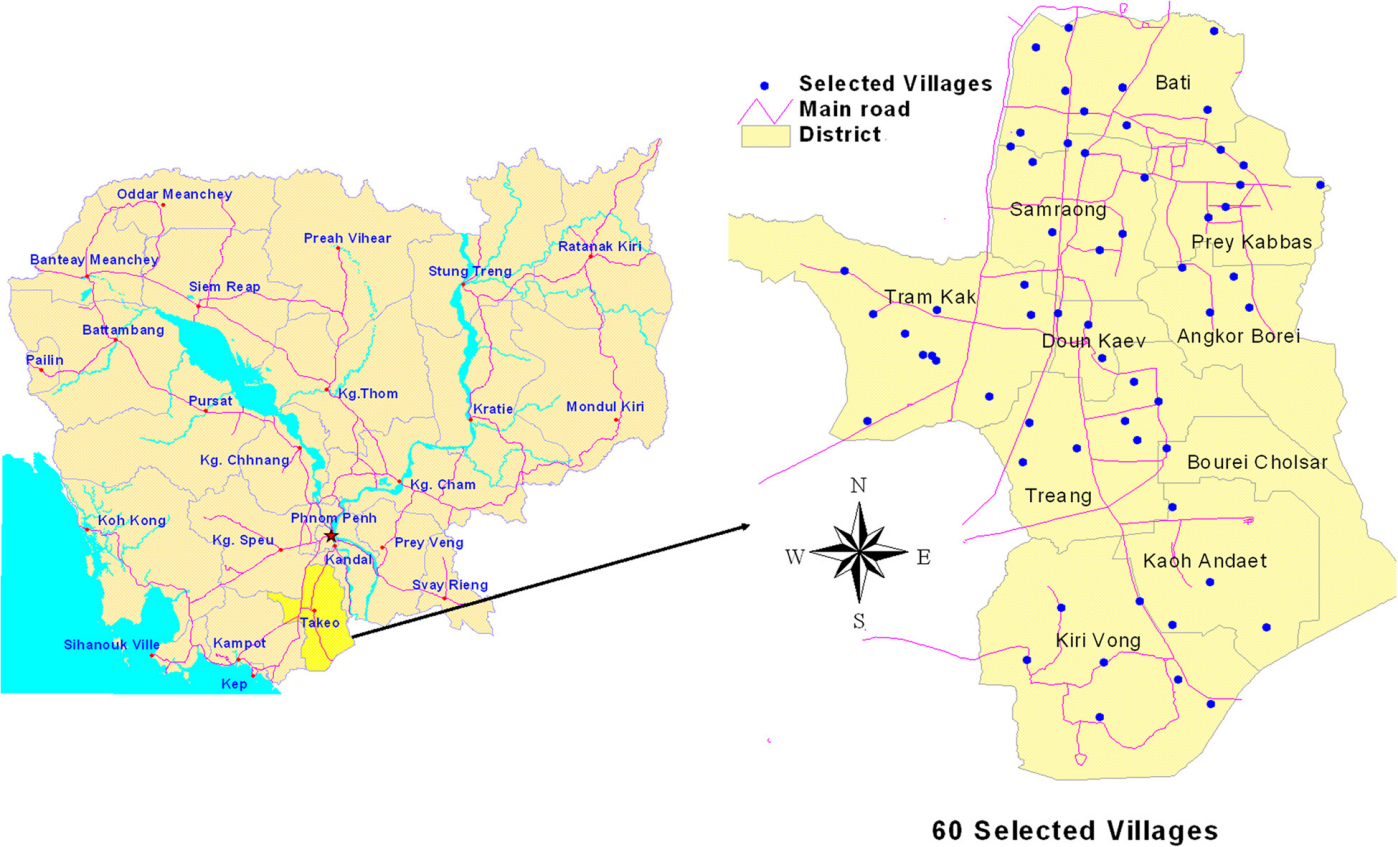
**Map of selected villages in Takeo province, Cambodia.**

A cross-sectional study was carried out from January to April, 2011, among the general population living throughout 60 villages of Takeo province, southern Cambodia (Figure 1). The villages were randomly selected from a list of all villages in the 10 districts of Takeo province (total number of villages: 1,118).

Takeo province, with its expansive floodplains, is located in the South of Cambodia, about 80 kilometres from the capital, Phnom Penh, along the border with Vietnam. Takeo has 843,931 inhabitants, with a density of 237 people per square kilometre. The economy is based on agriculture, namely fisheries, rice and fruit cropping. Pigs, dogs, cats, poultry and cattle are the most common domestic animals [22]. No information on the prevalence of *S. stercoralis* is available for Takeo province. However, the prevalences of intestinal helminth infections have recently been reported in Ang Svay Chek villages, Prey Kabas district, and were 13.2% for hookworm and 47.5% for *Opisthorchis viverrini* and minute intestinal flukes together [23].

Fifteen households were randomly selected from each village. All household members, 12 months or older, were eligible and informed about the study. All household members present on the day of the survey were enrolled.

### Field procedures

A pre-tested household questionnaire was administered to the head of household to obtain information on socioeconomic indicators such as house type (concrete, wood or leaves), household assets (sewing machine, television, radio, motorcycle or car), livestock (cattle, pigs or poultry) and availability of a latrine at home. Participants were interviewed based on a pre-tested individual questionnaire in order to collect information about demographics (age, gender, educational level and profession), personal risk-perception (knowledge about worm infections) personal hygiene practices (washing hands after defecating or before eating), wearing shoes (at home/work) and use of latrines for defecation. All questionnaires were developed in English and translated into Khmer. A different person translated the questionnaires back into English to ensure correct content. The interviews were conducted in Khmer language. After the interview, each participant received a pre-labelled plastic container (ID code, name, sex, age and date) for collecting a stool sample the next morning. Within two hours of collection, the stool specimens were sent (at ambient temperature) to the laboratory in Takeo town, where they were immediately processed.

### Laboratory procedures

First, two Kato-Katz thick smears were prepared, using the World Health Organization’s (WHO’s) standard template [24]. After a clearance time of 30 minutes, the smears were examined under a light microscope for the presence of helminth eggs. These were counted and recorded for each helminth species separately. Second, the KAP culture was used to identify *S. stercoralis* and hookworm larvae [13]. A hazelnut-sized stool sample was placed in the middle of the agar plate and the closed Petri dish was incubated in a humid chamber for 48 hours at 28°C. Then, the plate was rinsed with sodium acetate-acetic acid-formalin (SAF) solution. The eluent was centrifuged and the sediment microscopically examined for the presence of *S. stercoralis* and hookworm larvae. Finally, the Baermann technique was performed [12]. A walnut-sized stool sample was placed on gauze inserted into a glass funnel, and covered with tap water. The apparatus was exposed for two hours to artificial light directed from below. After centrifugation of the collected liquid, the sediment was examined under a microscope for the presence of *S. stercoralis* larvae.

For quality control, the technicians were specifically trained on the morphological criteria for distinguishing hookworm and *S. stercoralis* larvae. During the entire study period, a qualified microscopist from the Swiss Tropical and Public Health Institute, Basel, Switzerland, provided continuous and rigorous supervision. Further, any unclear diagnosis was immediately discussed with a qualified microscopist and the study supervisor.

### Data management and statistical analyses

Questionnaire and laboratory data collected from each participant was double-entered and validated in EpiData version 3.1 (EpiData Association; Odense, Denmark). Statistical analyses were performed with STATA version 12.1 (StataCorp.; College Station, TX, USA). Only participants with complete records (stool sample examined with all diagnostic methods and completed questionnaires) were included in the final analysis. A ‘smoothed’ age prevalence curve was used to present the prevalence distribution of the mean age of each participant.

Principle component analysis (PCA) was applied to variables pertaining to ownership of various household assets, and was used to build the socioeconomic status (SES) profile. SES was categorized by one of three wealth levels: least poor, less poor and poor, as previously described in detail [25].

Generalized Estimating Equations (GEE) were used to determine the association between infection status and demographic variables, hygienic status, knowledge and the recent medical history of participants. Variables with an odds ratio below 0.80 and above 1.25 in the bivariate models were selected for inclusion in the multivariate GEE model.

## Results

### Study population and compliance

In total, 3,568 individuals from 900 households (median household size: 5 members, range: 1 – 12) were enrolled, of which 3,154 (88.4%) submitted a stool sample. The final analysis included 2,861 (80.2%) participants with complete data records, i.e., enough stool for all three diagnostic tests (two Kato-Katz, KAP culture and Baermann method) and all questionnaire data.

Of the 2,861 participants with complete data records, 1,560 (54.5%) were female. The median age of all participants was 26 years, with a range from 1 to 90 years and 57.2% aged 30 years or under. One third or 31.7% were schoolchildren; 40.5% were farmers; three-quarters or 74.7% had attended school, with 50.5% and 24.2% attending primary and secondary school or higher, respectively.

### Parasitological findings and performance of diagnostic methods

**Figure 2:**
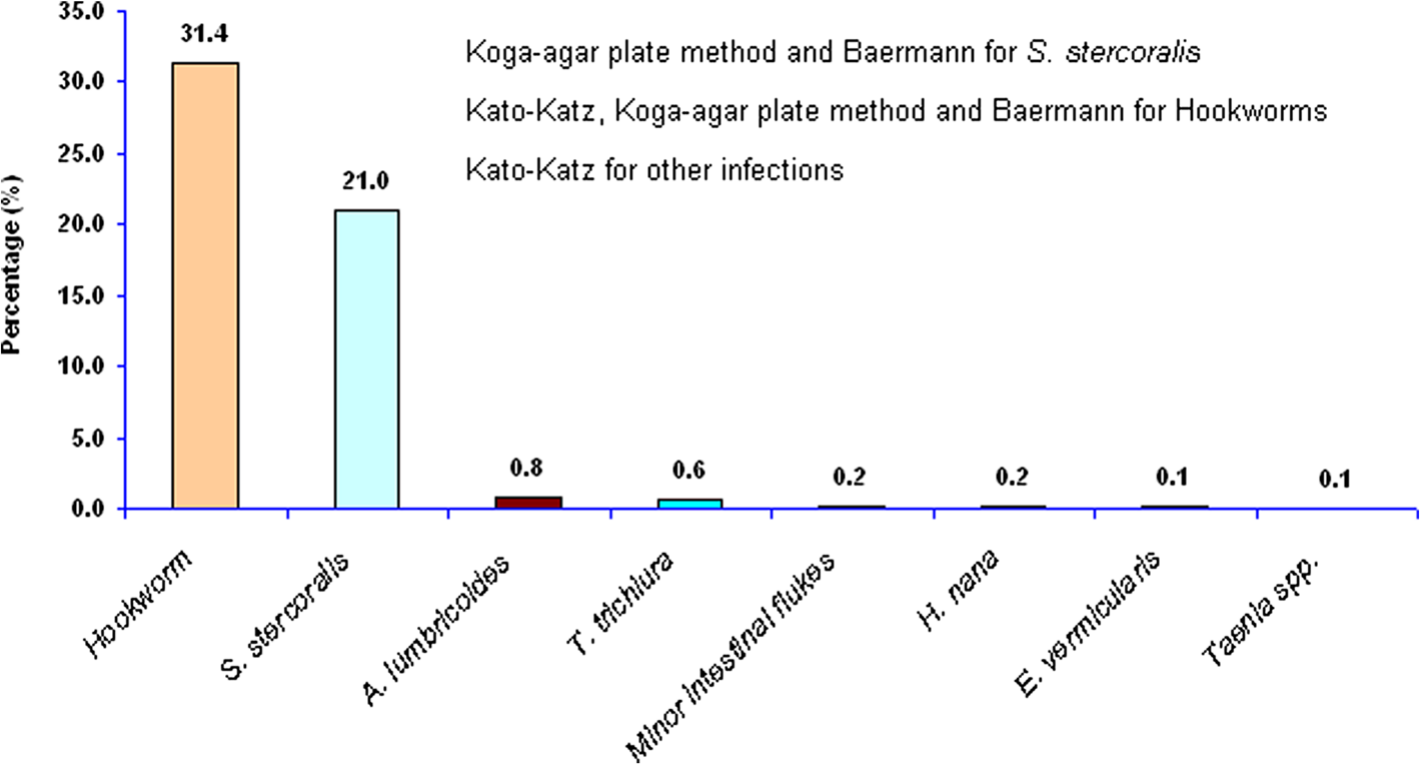
**Frequency of intestinal parasitic infections among 2,861 residents in Takeo province, Cambodia.**

Figure 2 shows the eight intestinal helminth species found among the participants. Hookworm and *S. stercoralis* were most frequent, with 31.4% and 21.0%, respectively. *Ascaris lumbricoides*, *Trichuris trichiura*, eggs of minor intestinal flukes (MIF), *Hymenolepis nana*, *Enterobius vermicularis* and *Taenia* spp. were detected, but with prevalences of less than 1%. Of 601 *S. stercoralis* cases, 46.9% were co-infected with hookworms.

Detailed information on the diagnostic performance of the Baermann technique and KAP culture is shown in Table 1. When combining the two methods as a “diagnostic gold standard”, the sensitivity of KAP culture and Baermann technique was 78.5% and 67.1%, respectively, with negative predictive values of 94.6% and 91.9%.

**Table 1:** **Yield of Koga**-**agar culture and Baermann method for detecting *S. stercoralis *** **in 2**,**861 participants**, **Takeo province**, **Cambodia**

		**Combined Methods (KAP culture and Baermann)**		**Total**
		**Positive**	**Negative**	
**KAP culture**	**Positive**	472	0	472
	**Negative**	129	2260	2389
	**Total**	601	2260	2861
**Baermann Technique**	**Positive**	403	0	403
	**Negative**	198	2260	2458
	**Total**	601	2260	2861

### Risk factors for *Strongyloides stercoralis* infection

**Figure 3:**
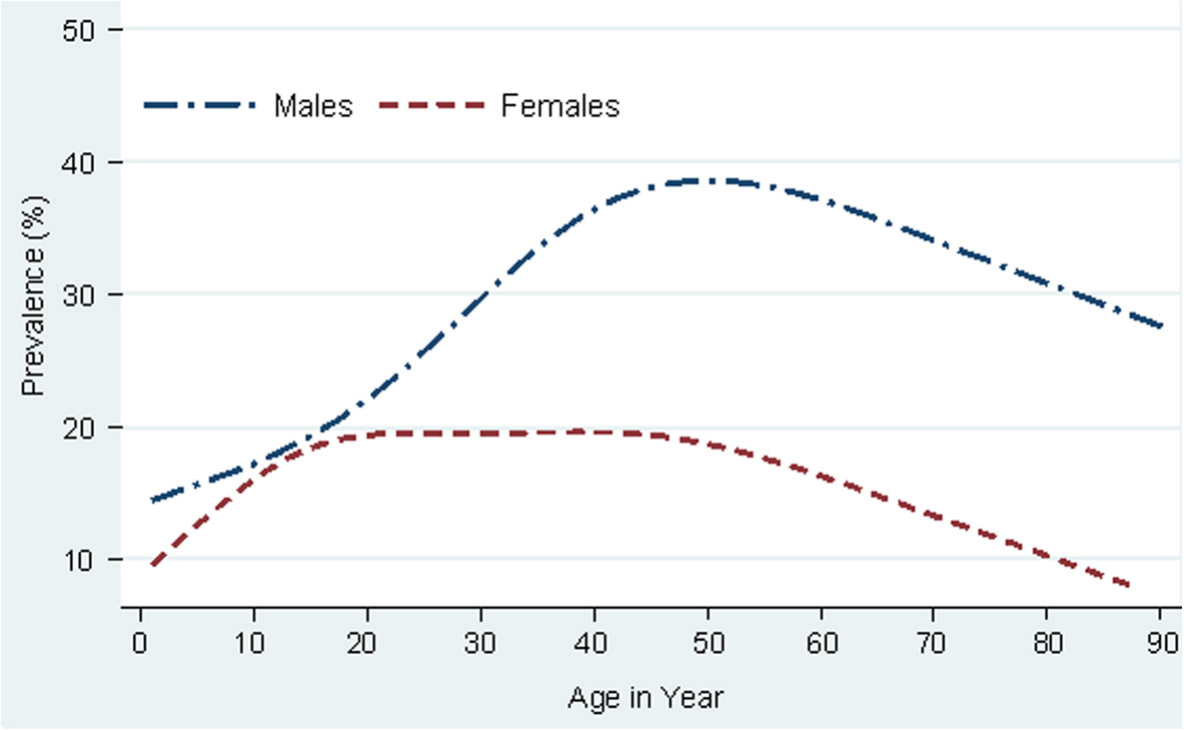
**Age prevalence of *S. stercoralis *** **infection by sex among 2,861 residents in Takeo province, Cambodia, 2011.**

A total of 16 risk factor variables were tested to identify the univariate association between *S. stercoralis* infection and socio-demographic status (6 variables), personal disease perception (4 variables) and hygiene data (6 variables). Table 2 contains 10 variables that were included in the multivariable analysis. The most important risk factors associated with *S. stercoralis* infection were gender and possession of a latrine at home. Males had a significantly higher risk of *S. stercoralis* infection than females (OR: 1.7; 95% CI: 1.4 – 2.0; *P* < 0.001), while participants with a latrine at home were less frequently infected than those who did not (OR: 0.7; 95% CI: 0.4 – 0.8; *P* = 0.003). The prevalence of *S. stercoralis* increased with age for both sexes (Figure 3), starting from 14.5% in children five years and under to a peak of 28.0% in individuals aged 56 to 60 years. In all age groups, males displayed a higher infection rate than females. Age, profession, education level and SES were not statistically different between non-infected and infected individuals.

**Table 2:** **Risk factors for *S*. *stercoralis *** **infection among 2,861 participants, Takeo province, Cambodia**

***Multivariable model***	**Non**-***S. stercoralis *** **(N** **=** **2260) n (%)**	***S. stercoralis *** **(N** **=** **601) n (%)**	**OR (95% CI)**	**p-Value**
**Demographic information**				
Gender (male)	969 (42.9)	332 (55.2)	1.7 (1.4 – 2.0)	<0.001
Age group				
1 - 15 years	868 (38.4)	168 (28.0)	Reference	
16 - 30 years	471 (20.8)	129 (21.5)	1.2 (0.8 - 1.7)	0.378
31 - 45 years	386 (17.1)	127 (21.1)	1.3 (0.9 - 1.9)	0.229
> 46 years	535 (23.7)	177 (29.4)	1.3 (0.9 - 2.0)	0.145
Profession				
Farmer/Rice-Grower	851 (37.7)	308 (51.3)	Reference	
Schoolchildren	752 (33.3)	154 (25.6)	0.7 (0.5 - 1.1)	0.097
Others	657 (29.1)	139 (23.1)	0.7 (0.5 - 0.9)	0.006
Socio-economic status				
Poor	631 (27.9)	195 (32.4)	Reference	
Less poor	745 (32.9)	185 (30.8)	0.8 (0.6 - 1.0)	0.092
Least poor	884 (39.1)	221 (36.8)	0.9 (0.7 - 1.2)	0.523
**Personal disease perception**				
Have been treated for worms (yes)	1249 (55.3)	269 (44.8)	0.8 (0.7 - 1.0)	0.055
Know about worms/infection with worms (yes)	933 (41.3)	277 (46.1)	1.0 (0.8 - 1.2)	0.890
**Personal hygiene**				
Toilet at home (yes)	949 (42.0)	192 (31.9)	0.7 (0.4 - 0.8)	0.003
Usually defecated in toilet (yes)	941 (41.6)	209 (34.8)	1.2 (0.8 - 1.7)	0.351
Had shoes (yes)	1982 (87.7)	542 (90.2)	1.1 (0.7 - 1.9)	0.697
Wore shoes when going to defecate/toilet (yes)	1846 (82.7)	514 (85.5)	1.1 (0.7 - 1.6)	0.786

OR: Odd Ratio; 95% CI: 95% Confident Interval.

### Characteristics of *Strongyloides stercoralis* cases

Of the 601 *S. stercoralis* cases, 332 (55.2%) were male and 308 (51.3%) were farmers. Almost one-third (28.0%) of the cases were children under 16 years, while 256 (42.6%) were between the ages of 16 and 45 years. A quarter (25.6%) of the cases occurred among schoolchildren (Table 2).

The medical problems reported during the two weeks prior to diagnoses are shown in Table 3. Muscle pain (OR: 1.3; 95% CI: 1.0 - 1.6; *P* = 0.028) and cutaneous rash (urticaria) (OR: 1.4; 95% CI: 1.1 - 1.8; *P* = 0.001) were significantly more frequent among those with *S. stercoralis* infection. No other reported clinical symptom was associated with *S. stercoralis* infection.

**Table 3:** **Clinical symptoms for *S. stercoralis *** **cases in 2**,**861 participants**, **Takeo province**, **Cambodia**, **2011**

**Symptoms mentioned in the past 2 weeks**	**Non-*S. stercoralis *** **(N** **=** **2260) n (%)**	***S. stercoralis *** **(N** **=** **601) n (%)**	**OR (95% CI)**	**p-Value**
Anorexic and/or loss of appetite (yes)	410 (18.1)	115 (19.1)	1.0 (0.8 - 1.3)	0.785
Sensation of tiredness (yes)	532 (23.5)	154 (25.6)	1.1 (0.9- 1.3)	0.492
Nausea (yes)	307 (13.6)	80 (13.3)	1.0 (0.8 - 1.3)	0.925
Vomiting (yes)	286 (12.7)	63 (10.5)	0.8 (0.6 - 1.1)	0.180
Diarrhoea (yes)	753 (33.3)	204 (33.9)	1.0 (0.9 - 1.3)	0.677
Bloody diarrhoea (yes)	83 (3.7)	22 (3.7)	1.0 (0.6 - 1.7)	0.852
Greasy diarrhoea (yes)	177 (7.8)	50 (8.3)	1.0 (0.7 - 1.4)	0.855
Constipation (yes)	305 (13.5)	63 (10.5)	0.8 (0.6 - 1.0)	0.088
Itching (yes)	822 (36.4)	225 (37.4)	1.1 (0.9 - 1.3)	0.334
Seen worm in stool (yes)	215 (9.5)	45 (7.5)	0.8 (0.6 - 1.1)	0.278
Cough (yes)	1308 (57.9)	351 (58.4)	1.1 (0.9 - 1.3)	0.552
Coughed out a worm (yes)	11 (0.5)	2 (0.3)	0.7 (0.2 - 3.0)	0.625
Cutaneous rash “urticaria” (yes)	397 (17.6)	138 (23.0)	1.4 (1.1 - 1.8)	0.001
Abdominal pain (yes)	1121 (49.6)	278 (46.3)	0.9 (0.8 - 1.1)	0.300
Muscle pain (yes)	360 (15.9)	118 (19.6)	1.3 (1.0 - 1.6)	0.028
Lost weight (yes)	310 (13.7)	90 (15.0)	1.1 (0.8 - 1.4)	0.578

OR: Odd Ratio; 95% CI: 95% Confident Interval.

## Discussion

The worldwide distribution of *S. stercoralis* infection varies greatly from country to country and even within the same country, depending on ecological and socioeconomic conditions [5]. *S. stercoralis* infection is commonly overlooked, especially in endemic resource poor countries, as the most suitable diagnostic methods for *S. stercoralis* (KAP culture and Baermann technique) are not used in most epidemiological studies of soil-transmitted helminths [1,5]. Despite the high endemicity of *S. stercoralis* in Southeast Asia, specific information is often lacking. Thailand is the only country in the region with a considerable amount of information available on *S. stercoralis* prevalence [5]. Our community-based study of *S. stercoralis* infection among 2,861 participants from 60 villages in a southern province of Cambodia, employing KAP culture and the Baermann method, found a prevalence of 21.0%.

To our knowledge, this is the first community-based report in Cambodia on the importance of *S. stercoralis* infection in a large province-wide setting, using a rigorous diagnostic technique (KAP culture and the Baermann technique). Recent surveys on *S. stercoralis* infection in Cambodia studied mainly the diagnosis, treatment and risk factors of infection among schoolchildren [6], the clinical aspects of high intensity infection [18] and the molecular diagnostic approach (PCR) [11]. Our report highlights the high frequency of the parasite in the general population, and points to key risk factors, which need to be addressed in large-scale control programmes (country-wide).

Our observed prevalence is particularly high compared to those of two previous studies that used the Baermann technique on samples from school-aged children (12.0%) and from the general population in Cambodia (14.6%) [8,9]. One possible explanation is that the present study used a combined diagnostic method (KAP culture and Baermann technique) to diagnose *S. stercoralis*, while others used a single test. However, a similar prevalence rate (20.2%) was observed in a 2006 study that had only used the Baermann technique to analyse a single stool sample from school-aged children living in villages bordering Tonlé Sap Lake, central-northern Cambodia [7]. Moreover, a recent study among schoolchildren in Kandal province, central-southern Cambodia, which used the same laboratory approach on multiple stool samples, reported a prevalence of 24.4% [6], which is consistent with our findings when using the same diagnostic methods on a single stool sample. This observation suggests that the overall prevalence of *S. stercoralis* in our study population would actually have been higher if several stool samples had been examined.

The prevalence of *S. stercoralis* in our study setting is higher than those shown in other studies among the general population in Southeast Asian countries: Lao PDR, Thailand and China. In Lao PDR, the study conducted by Sayasone *et al*. in 2009, using a formalin ethyl-acetate concentration technique on a single stool sample, found that 10.3% of participants were infected with *S. stercoralis*, but this diagnostic approach is not very sensitive [26]. In Thailand, Nontasut *et al*. used KAP culture to analyse a single stool sample and reported a prevalence of 15.9% [27]. In China, Steinmann *et al*. examined three stool samples by KAP culture and Baermann method and found a prevalence of 11.7% [14].

In our study, the observed prevalence of *S. stercoralis* was significantly higher in males than in females, which coincides with previous reports from Thailand [27] and China [14]. In addition, males had a higher infection rate than females in all age groups. This is likely due to agricultural practices and activities. Most men are rice farmers and work in muddy rice fields without footwear. In contrast, Cambodian females usually work as housewives and wear shoes when walking around the household or village. The prevalence of *S. stercoralis* also increased with age, starting from 14.5% in children 5 years and under, to a peak prevalence of 28.0% in participants aged 56 to 60, decreasing slightly thereafter. This suggests that infection first occurs in the village or household where young children play, usually without shoes. After contracting the parasite at a young age, the infection may remain for decades in the host if the infection is not treated [28-30], which would account for the increased prevalence associated with age.

The clinical manifestations of strongyloidiasis vary greatly between immune-competent and immune-suppressed individuals. Gastrointestinal (nausea and diarrhea) and cutaneous (itchiness and urticaria) symptoms are frequently described. However, more than 50% of strongyloidiasis cases are asymptomatic [18,19,31,32]. In our study, we observed that muscle pain and urticaria reported during the preceding two weeks were associated with *S. stercoralis* infection; a finding that coincides with recent reports from the Northern province of Cambodia [18]. The clinical features of strongyloidiasis and the association between strongyloidiasis and other infectious diseases are not well understood [19,31]. With the limitations of our study, it was not possible to differentiate underlying infections that might mimic strongyloidiasis. Therefore, an indepth assessment on the clinical symptoms of *S. stercoralis* infection is required.

The sensitivity of KAP culture and the Baermann technique has been reviewed in several studies, with contradictory results. Our study found the sensitivity of KAP culture to be higher than that of the Baermann method, contrary to previous reports from south-central Côte d’Ivoire [33], Zanzibar [34], Uganda [35] and China [14]. However, our findings are consistent with those reported in studies conducted in Cambodia [6], Brazil [16], rural Côte d’Ivoire [15] and Honduras [36].

Our study examined only a single stool sample per participant, thus the prevalence of *S. stercoralis* in our study setting was likely under-estimated, since the excretion of *S. stercoralis* larvae in stool specimens varies considerably from day-to-day [34,37]. A recent study showed that the output of larvae in faecal specimens ranged from 0.003 larvae per gram to 151.2 larvae per gram, as observed over seven consecutive days [37]. However, even when multiple stool samples are available, no single diagnostic test, KAP culture or Baermann method, can detect all *S. stercoralis* infection [6,34]. Therefore, the combined use of both diagnostic methods (KAP culture and Baermann technique) on several stool specimens will increase sensitivity and currently represents the best diagnostic approach for this infection.

## Conclusions

*S. stercoralis* infection is highly prevalent among the general population in Cambodia and should be given more attention due to its potential for disseminating infection. Access to adequate diagnosis and treatment is urgently required in Cambodia.

## Competing interests

The authors declare that they have no competing interest.

## Authors’ contributions

VK, FS, SM and PO conceived and designed the study. VK collected field data and FS and PJB analysed the stool specimens. HM coordinated the laboratory activities. MCC and SM coordinated the field work in Cambodia. VK analysed the data and wrote the manuscript together with PO. PO supervised the first author in all aspects of the study. All authors have read and approved the final version of manuscript.

## References

[B1] Olsen A van Lieshout L Marti H Polderman T Polman K Steinmann P Stothard R Thybo S Verweij JJ Magnussen P Strongyloidiasis–the most neglected of the neglected tropical diseases? Trans R Soc Trop Med Hyg 2009 103 10 967 972 10.1016/j.trstmh.2009.02.013 19328508

[B2] Bethony J Brooker S Albonico M Geiger SM Loukas A Diemert D Hotez PJ Soil-transmitted helminth infections: ascariasis, trichuriasis, and hookworm Lancet 2006 367 9521 1521 1532 10.1016/S0140-6736(06)68653-4 16679166

[B3] Paula FM Costa-Cruz JM Epidemiological aspects of strongyloidiasis in Brazil Parasitology 2011 138 11 1331 1340 10.1017/S003118201100120X 21810305

[B4] Requena-Mendez A Chiodini P Bisoffi Z Buonfrate D Gotuzzo E Munoz J The laboratory diagnosis and follow up of strongyloidiasis: a systematic review PLoS Negl Trop Dis 2013 7 1 e2002 10.1371/journal.pntd.0002002 23350004 PMC3547839

[B5] Schär F Trostdorf U Giardina F Khieu V Muth S Marti H Vounatsou P Odermatt P *Strongyloides stercoralis*: Global Distribution and Risk Factors PLoS Negl Trop Dis 2013 7 7 e2288 10.1371/journal.pntd.0002288 23875033 PMC3708837

[B6] Khieu V Schär F Marti H Sayasone S Duong S Muth S Odermatt P Diagnosis, Treatment and Risk Factors of *Strongyloides stercoralis* in Schoolchildren in Cambodia PLoS Negl Trop Dis 2013 7 2 e2035 10.1371/journal.pntd.0002035 23409200 PMC3566990

[B7] Chhakda T Muth S Socheat D Odermatt P Intestinal parasites in school-aged children in villages bordering Tonle Sap Lake, Cambodia Southeast Asian J Trop Med Public Health 2006 37 5 859 864 17333726

[B8] Koga-Kita K Intestinal parasitic infections and socioeconomic status in Prek Russey Commune, Cambodia [Nihon koshu eisei zasshi] Japanese J Public Health 2004 51 11 986 992 15678991

[B9] Longfils P Heang UK Soeng H Sinuon M Weekly iron and folic acid supplementation as a tool to reduce anemia among primary school children in Cambodia Nutr Rev 2005 63 12 Pt 2 S139 S145 16466090 10.1301/nr.2005.dec.s139-s145

[B10] Moore CE Hor PC Soeng S Sun S Lee SJ Parry CM Day NP Stoesser N Changing Patterns of Gastrointestinal Parasite Infections in Cambodian Children: 2006-2011 J Trop Pediatr 2012 58 6 509 512 10.1093/tropej/fms024 22723077 PMC3739457

[B11] Schär F Odermatt P Khieu V Panning M Duong S Muth S Marti H Kramme S Evaluation of real-time PCR for *Strongyloides stercoralis* and hookworm as diagnostic tool in asymptomatic schoolchildren in Cambodia Acta Trop 2013 126 2 89 92 10.1016/j.actatropica.2012.12.012 23298731

[B12] Garcia L Bruckner D Diagnostic medical parasitology 2001 eds Washington DC: American Society for Microbiology 1 179

[B13] Koga K Kasuya S Khamboonruang C Sukhavat K Ieda M Takatsuka N Kita K Ohtomo H A modified agar plate method for detection of *Strongyloides stercoralis* Am J Trop Med Hyg 1991 45 4 518 521 1951861 10.4269/ajtmh.1991.45.518

[B14] Steinmann P Zhou XN Du ZW Jiang JY Wang LB Wang XZ Li LH Marti H Utzinger J Occurrence of *Strongyloides stercoralis* in Yunnan Province, China, and comparison of diagnostic methods PLoS Negl Trop Dis 2007 1 1 e75 10.1371/journal.pntd.0000075 17989788 PMC2041812

[B15] Glinz D N'Guessan NA Utzinger J N’Goran EK High prevalence of *Strongyloides stercoralis* among school children in rural Cote d’Ivoire J Parasitol 2010 96 2 431 433 10.1645/GE-2294.1 19916629

[B16] Ines Ede J Souza JN Santos RC Souza ES Santos FL Silva ML Silva MP Teixeira MC Soares NM Efficacy of parasitological methods for the diagnosis of *Strongyloides stercoralis* and hookworm in faecal specimens Acta Trop 2011 120 3 206 210 10.1016/j.actatropica.2011.08.010 21896267

[B17] Getaneh A Medhin G Shimelis T *Cryptosporidium* and *Strongyloides stercoralis* infections among people with and without HIV infection and efficiency of diagnostic methods for *Strongyloides* in Yirgalem Hospital, southern Ethiopia BMC research notes 2010 3 90 10.1186/1756-0500-3-90 20359359 PMC2873353

[B18] Khieu V Srey S Schär F Muth S Marti H Odermatt P *Strongyloides stercoralis* is a cause of abdominal pain, diarrhea and urticaria in rural Cambodia BMC Res Notes 2013 6 1 200 10.1186/1756-0500-6-200 23688049 PMC3668207

[B19] Vadlamudi RS Chi DS Krishnaswamy G Intestinal strongyloidiasis and hyperinfection syndrome Clin Mol Allergy 2006 4 8 10.1186/1476-7961-4-8 16734908 PMC1538622

[B20] Marti H Haji HJ Savioli L Chwaya HM Mgeni AF Ameir JS Hatz C A comparative trial of a single-dose ivermectin versus three days of albendazole for treatment of *Strongyloides stercoralis* and other soil-transmitted helminth infections in children Am J Trop Med Hyg 1996 55 5 477 481 8940976 10.4269/ajtmh.1996.55.477

[B21] CNM National Policy and Guideline for Helminth Control in Cambodia 2004 Phnom Penh, Cambodia: National Center for Parasitology, Entomology and Malaria Control, Ministry of Health

[B22] NIS General Population Census of Cambodia 2008 2008 Phnom Penh, Cambodia: National Institute of Statistic, Ministry of Plan

[B23] Yong TS Shin EH Chai JY Sohn WM Eom KS Lee DM Park K Jeoung HG Hoang EH Lee YH Woo HJ Lee JH Kang SI Cha JK Lee KH Yoon CH Sinuon M Socheat D High prevalence of *Opisthorchis viverrini* infection in a riparian population in Takeo Province, Cambodia Korean J Parasitol 2012 50 2 173 176 10.3347/kjp.2012.50.2.173 22711932 PMC3375459

[B24] Katz N Chaves A Pellegrino J A simple device for quantitative stool thick-smear technique in *Schistosomiasis mansoni* Rev Inst Med Trop Sao Paulo 1972 14 6 397 400 4675644

[B25] Vyas S Kumaranayake L Constructing socio-economic status indices: how to use principal components analysis Health Policy Plan 2006 21 6 459 468 10.1093/heapol/czl029 17030551

[B26] Sayasone S Vonghajack Y Vanmany M Rasphone O Tesana S Utzinger J Akkhavong K Odermatt P Diversity of human intestinal helminthiasis in Lao PDR Trans R Soc Trop Med Hyg 2009 103 3 247 254 10.1016/j.trstmh.2008.10.011 19038411

[B27] Nontasut P Muennoo C Sa-nguankiat S Fongsri S Vichit A Prevalence of *Strongyloides* in Northern Thailand and treatment with ivermectin vs albendazole Southeast Asian J Trop Med Public Health 2005 36 2 442 444 15916052

[B28] Prendki V Fenaux P Durand R Thellier M Bouchaud O Strongyloidiasis in man 75 years after initial exposure Emerg Infect Dis 2011 17 5 931 932 10.3201/eid1705.100490 21529417 PMC3321755

[B29] Concha R Harrington W Jr Rogers AI Intestinal strongyloidiasis: recognition, management, and determinants of outcome J Clin Gastroenterol 2005 39 3 203 211 10.1097/01.mcg.0000152779.68900.33 15718861

[B30] Montes M Sawhney C Barros N *Strongyloides stercoralis*: there but not seen Curr Opin Infect Dis 2010 23 5 500 504 10.1097/QCO.0b013e32833df718 20733481 PMC2948977

[B31] Ly MN Bethel SL Usmani AS Lambert DR Cutaneous *Strongyloides stercoralis* infection: an unusual presentation J Am Acad Dermatol 2003 49 2 Suppl Case Reports S157 S160 12894109 10.1067/mjd.2003.338

[B32] Koczka CP Hindy P Goodman A Gress F Strongyloidiasis: a diagnosis more common than we think Eur J Gastroenterol Hepatol 2012 24 7 860 862 10.1097/MEG.0b013e3283543ea0 22555259

[B33] Becker SL Sieto B Silue KD Adjossan L Kone S Hatz C Kern WV N’Goran EK Utzinger J Diagnosis, clinical features, and self-reported morbidity of *Strongyloides stercoralis* and hookworm infection in a Co-endemic setting PLoS Negl Trop Dis 2011 5 8 e1292 10.1371/journal.pntd.0001292 21886853 PMC3160297

[B34] Knopp S Mgeni AF Khamis IS Steinmann P Stothard JR Rollinson D Marti H Utzinger J Diagnosis of soil-transmitted helminths in the era of preventive chemotherapy: effect of multiple stool sampling and use of different diagnostic techniques PLoS Negl Trop Dis 2008 2 11 e331 10.1371/journal.pntd.0000331 18982057 PMC2570799

[B35] Stothard JR Pleasant J Oguttu D Adriko M Galimaka R Ruggiana A Kazibwe F Kabatereine NB *Strongyloides stercoralis*: a field-based survey of mothers and their preschool children using ELISA, Baermann and Koga plate methods reveals low endemicity in western Uganda J Helminthol 2008 82 3 263 269 18416881 10.1017/S0022149X08971996

[B36] de Kaminsky RG Evaluation of three methods for laboratory diagnosis of *Strongyloides stercoralis* infection J Parasitol 1993 79 2 277 280 10.2307/3283519 8459339

[B37] Schär F Hattendorf J Khieu V Muth S Char MC Marti HP Odermatt P *Strongyloides stercoralis* larvae excretion patterns before and after treatment Parasitology 2014 141 7 892 897 10.1017/S0031182013002345 24534076

